# Integrating Clinical Perspectives in the Prevention of Teenage Pregnancy

**DOI:** 10.1111/hex.70570

**Published:** 2026-01-14

**Authors:** Erhan Muluk

**Affiliations:** ^1^ Department of Obstetrics and Gynecology Lara Anatolia Hospital Antalya Turkey

I read with great interest the article by Malapela et al., titled “Exploring Stakeholder Roles and Strategies for Preventing Teenage Pregnancy: A Comprehensive Analysis in Lepelle‐Nkumpi District, South Africa” (2025) [1]. The authors make a very valuable contribution by foregrounding the voices of non‐governmental organizations (NGOs) working closely with teenagers in rural communities. Their thematic analysis, highlighting multi‐stakeholder collaboration, economic empowerment, nurturing family bonds, information sharing, and extramural activities offers a rich and pragmatic framework for community‐level action.

As an obstetrician working in teenager and maternal health, I would like to offer a complementary perspective relevant to this era. The study deliberately focused on NGO workers; however, obstetricians, midwives, and nurses are also central actors in both the prevention and clinical management of teenage pregnancy. These professionals are often the first to see pregnant teenagers, manage complications, and provide post‐pregnancy contraception. Their perspectives can offer valuable guidance for policymakers, communities, and stakeholders.

In my daily practice as an obstetrician, I have encountered teenagers presenting with complications of early pregnancy or with a second pregnancy within a short interval, despite having previously interacted with health services. Many of these young patients shared that no one had ever discussed effective contraception with them in a clear, nonjudgmental manner after their first pregnancy or abortion. This represents a missed opportunity for prevention. These experiences suggest that hospital‐ and clinic‐based providers are not only treating the consequences of teenage pregnancy but are also uniquely positioned to intervene and prevent repeat pregnancies through timely counselling.

In a study conducted in socioeconomically deprived neighborhoods with high immigrant populations, a culturally adapted community‐based contraceptive counselling program was implemented. Following the intervention, fertility rates among girls aged 15–19 years declined by approximately 40%. The authors concluded that brief, community‐based counselling initiatives can significantly reduce adolescent fertility rates [2]. It is likely that if midwives and nurses were assigned community‐based roles and supported to conduct such initiatives, similar reductions could be achieved.

Obstetricians can also allocate additional time within hospital settings to actively focus on preventing repeat pregnancies. The review by Sedlecky and Stanković emphasized that the most effective strategy to prevent repeated unintended adolescent pregnancies is to initiate an effective contraceptive method immediately after abortion. This timing is critical, as motivation for contraception is highest at that moment. Ovulation typically resumes within 3 weeks, and more than half of adolescents resume sexual activity within 2 weeks after pregnancy termination. The authors stressed that long‐acting reversible contraceptives such as intrauterine devices and implants are the most reliable options since their efficacy is not user‐dependent. However, because long‐term continuation can be challenging for adolescents, nonjudgmental, supportive counselling and regular follow‐ups are also essential [3].

There are additional ways to avoid repeat pregnancies if we can identify the teenagers at greatest risk. A systematic review and meta‐analysis by Maravilla et al. analyzed 26 epidemiological studies examining risk and protective factors for repeated teenage pregnancy. The findings showed that depression increases the likelihood of repeated pregnancy by 46% [4]. Therefore, during hospital or clinic visits, these girls would benefit from access to psychiatric and psychological support.

I fully agree with the authors that no single sector can address teenage pregnancy in isolation. My suggestion is not a criticism of their work but rather a proposal for future research and program design: to intentionally bring together NGOs, schools, churches, communities, and frontline sexual and reproductive health professionals (midwives, nurses, obstetricians), as well as psychiatrists and psychologists, in joint planning and evaluation. Such an integrated model could enhance both the reach and the effectiveness of the strategies so clearly described in this important study.

Another recommendation is that, whenever possible, health professionals providing counselling and contraceptive services to teenagers should preferably be female, as this may help young girls feel more comfortable asking sensitive questions. Although this advantage is less pronounced in obstetrics–gynecology compared to general medical practice, patients still tend to talk longer, share more biomedical and psychosocial information, and behave more assertively when interacting with female physicians [5].

In summary, teenagers should have opportunities to meet midwives and nurses in community settings to learn about contraception. If they become pregnant and are admitted to hospitals, physicians should preferably provide counselling and initiate contraceptive methods immediately after abortion, as this remains the most powerful strategy for preventing repeat pregnancies. Long‐acting contraceptives should be implemented where medically indicated, and it should not be forgotten that depression also needs to be addressed while the patient is still in the hospital setting.

Although I am aware that these recommendations may be difficult to implement especially in rural areas or in regions where access to health services is limited, drawing an ideal roadmap can still provide valuable guidance for policymakers, communities, and stakeholders.

## Author Contributions

**Erhan Muluk:** conceptualization, data curation, formal analysis, visualization, writing – original draft, writing – review and editing, project administration, investigation, methodology, software, validation, funding acquisition, resources.

## Funding

The author received no specific funding for this work.

## Conflicts of Interest

The author declares no conflicts of interest.

## Data Availability

The data that support the findings of this study are available from the corresponding author upon reasonable request.
